# Supplementary material for: Uremic Toxin Indoxyl Sulfate Promotes Macrophage-Associated Low-Grade Inflammation and Epithelial Cell Senescence
Source: Int J Mol Sci. 2023 Apr 28;24(9):8031. doi: 10.3390/ijms24098031 (PMC10179130; doi:10.3390/ijms24098031)
Supplement: Supplementary file 1 [file ijms-24-08031-s001.zip › ijms-2345636-supplementary/Supplementary Figure S1.pdf]

Figure S1

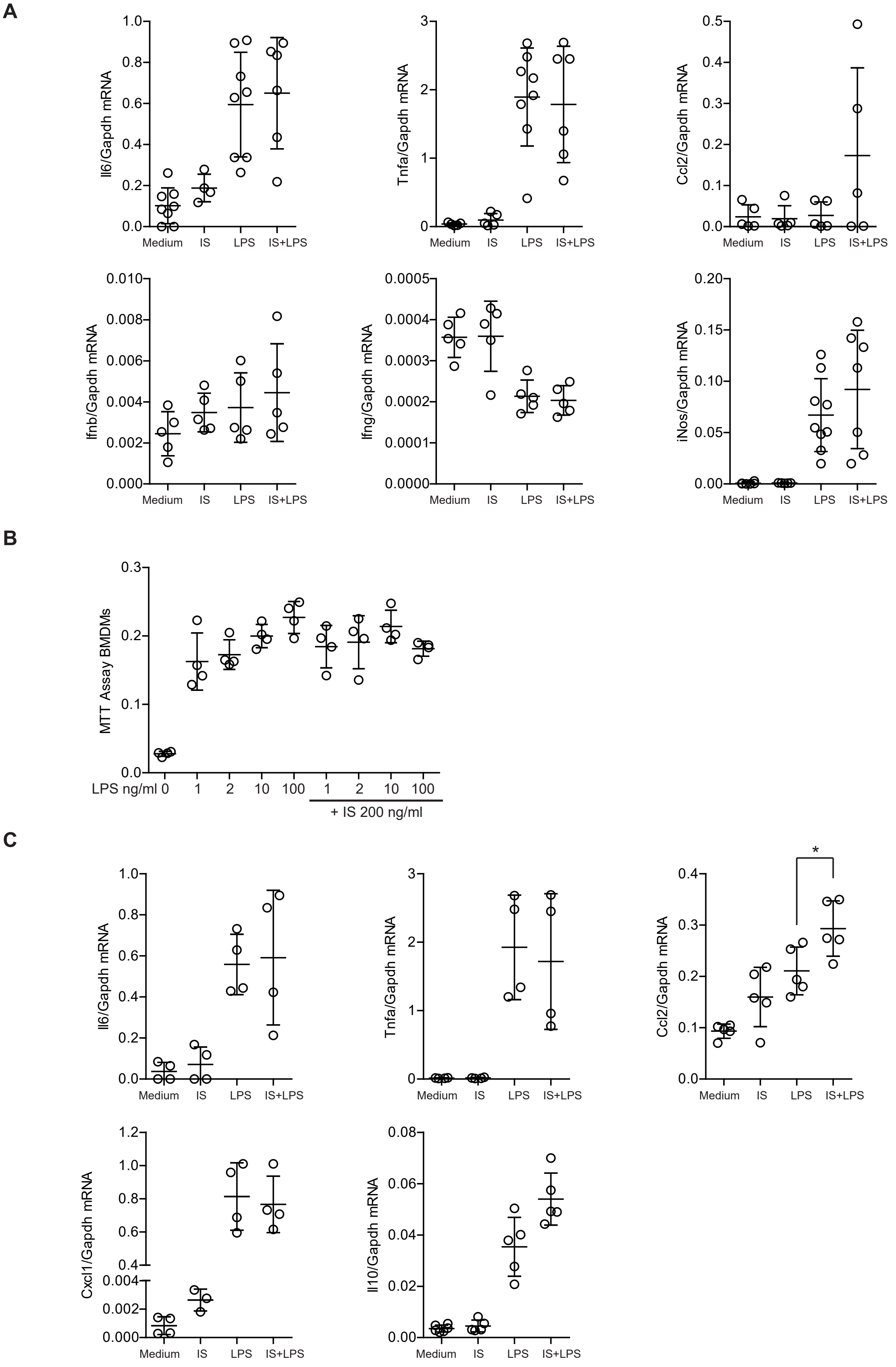

Figure S1. Low concentrations of IS do not significantly affect metabolic activity and inflammatory gene expression in BMDM (A) The mRNA expression levels of genes in bone marrow-derived macrophages (BMDM); we cultured the cells in medium containing 200 ng/ml indooxyl sulfate (IS) and stimulated them with LPS (2 ng/mL) for 4h.
(B) The viability and metabolic activity of BMDM was measured using MTT assay upon LPS stimulation (1-100 ng/mL) and in the absence and the abundance of IS. (C) The mRNA expression levels of genes in BMDM; we cultured the cells in medium containing 10 μg/ml indooxyl sulfate (IS) and stimulated them with LPS (2 ng/mL) for 4h.
Data are shown as means ± SD. * $p < 0.05$.
