# Supplementary material for: Uremic Toxin Indoxyl Sulfate Promotes Macrophage-Associated Low-Grade Inflammation and Epithelial Cell Senescence
Source: Int J Mol Sci. 2023 Apr 28;24(9):8031. doi: 10.3390/ijms24098031 (PMC10179130; doi:10.3390/ijms24098031)
Supplement: Supplementary file 1 [file ijms-24-08031-s001.zip › ijms-2345636-supplementary/Supplementary Figure S2.pdf]

Figure S2

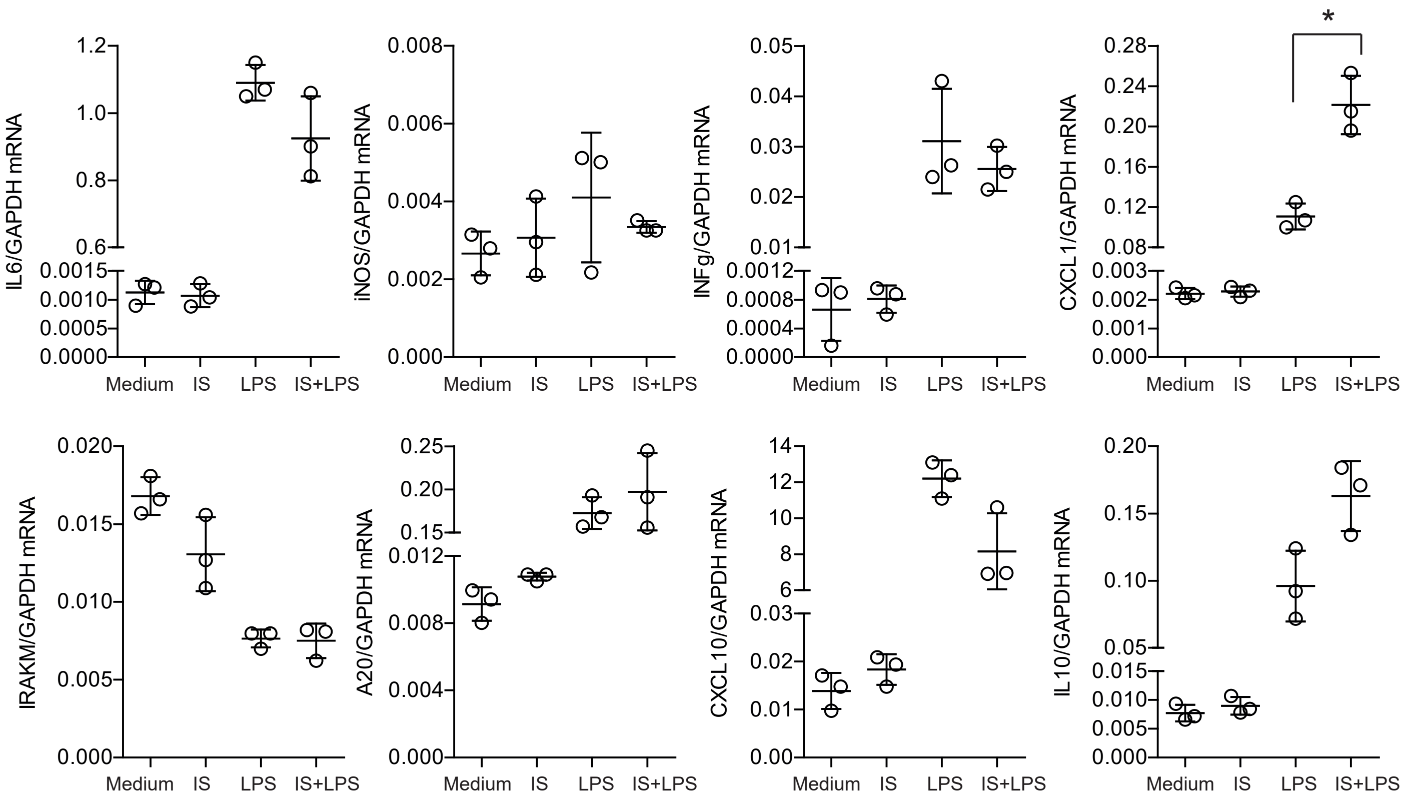

Figure S2. Indoxyl sulfate affect inflammatory gene expression in PBMCs. The mRNA expression levels of genes in PBMCs; we cultured the cells in medium containing 60μg/ml indooxyl sulfate (IS) and stimulated them with LPS (2 ng/mL) for 4h. Data are shown as means ± SD. * $p < 0.05$.
