# Supplementary material for: Uremic Toxin Indoxyl Sulfate Promotes Macrophage-Associated Low-Grade Inflammation and Epithelial Cell Senescence
Source: Int J Mol Sci. 2023 Apr 28;24(9):8031. doi: 10.3390/ijms24098031 (PMC10179130; doi:10.3390/ijms24098031)
Supplement: Supplementary file 1 [file ijms-24-08031-s001.zip › ijms-2345636-supplementary/Supplementary Figure S3.pdf]

Figure S3

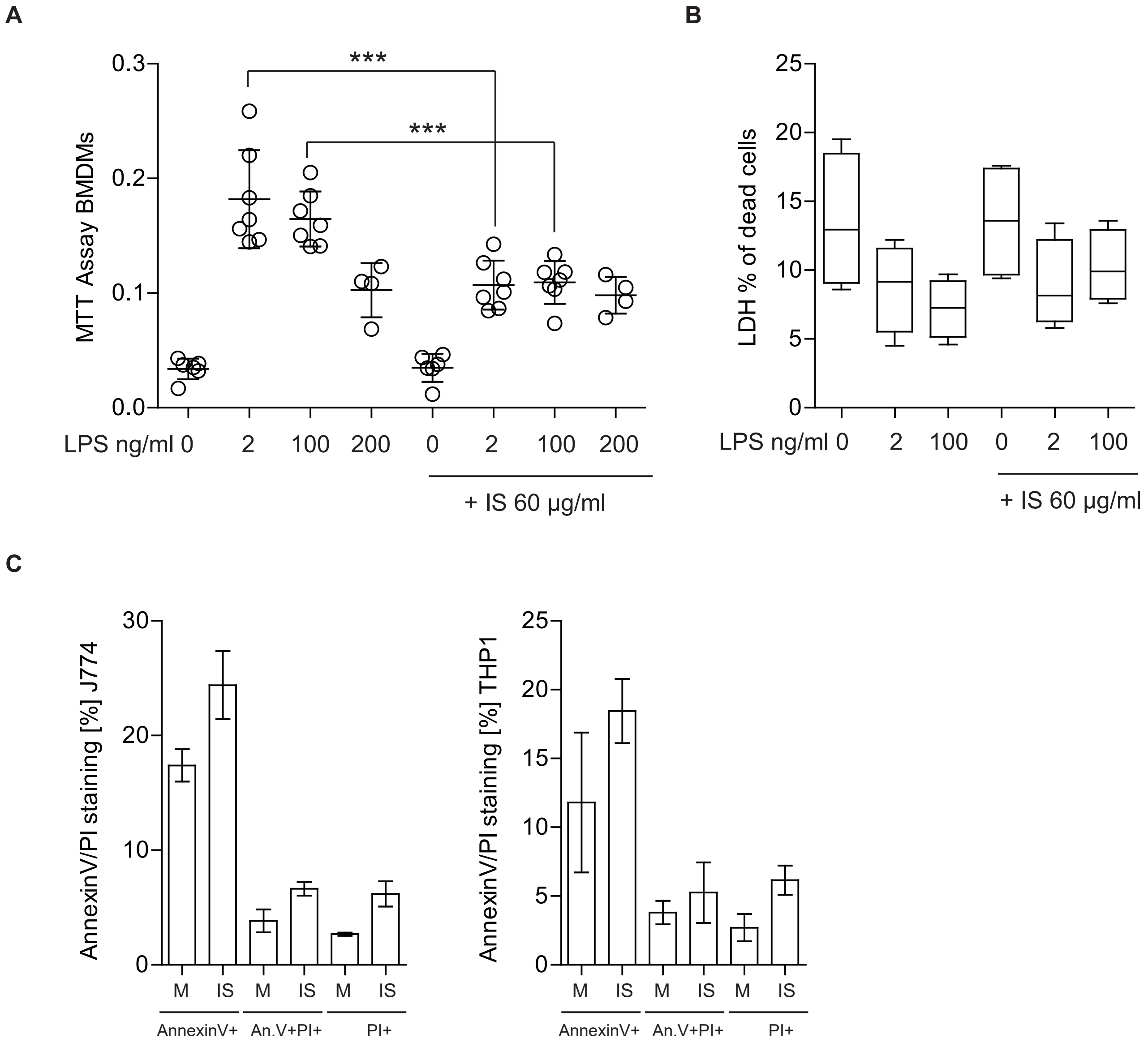

Figure S3. Indoxyl sulfate significantly inhibits metabolic activity but not the viability of BMDM. (A) The viability and metabolic activity of BMDM were measured using MTT assay upon LPS stimulation (2-200 ng/mL) and in the absence and abundance of IS. (B) LDH release was measured calorimetrically upon LPS stimulation (2-100 ng/mL) and in the absence and the abundance of IS. (C) The viability of J774 and THP1 cells were measured using flow cytometry in the absence and the abundance of IS. Data are shown as means ± SD; *** $p < 0.001$.
