# Supplementary material for: Uremic Toxin Indoxyl Sulfate Promotes Macrophage-Associated Low-Grade Inflammation and Epithelial Cell Senescence
Source: Int J Mol Sci. 2023 Apr 28;24(9):8031. doi: 10.3390/ijms24098031 (PMC10179130; doi:10.3390/ijms24098031)
Supplement: Supplementary file 1 [file ijms-24-08031-s001.zip › ijms-2345636-supplementary/Supplementary Figure S5.pdf]

Figure S5

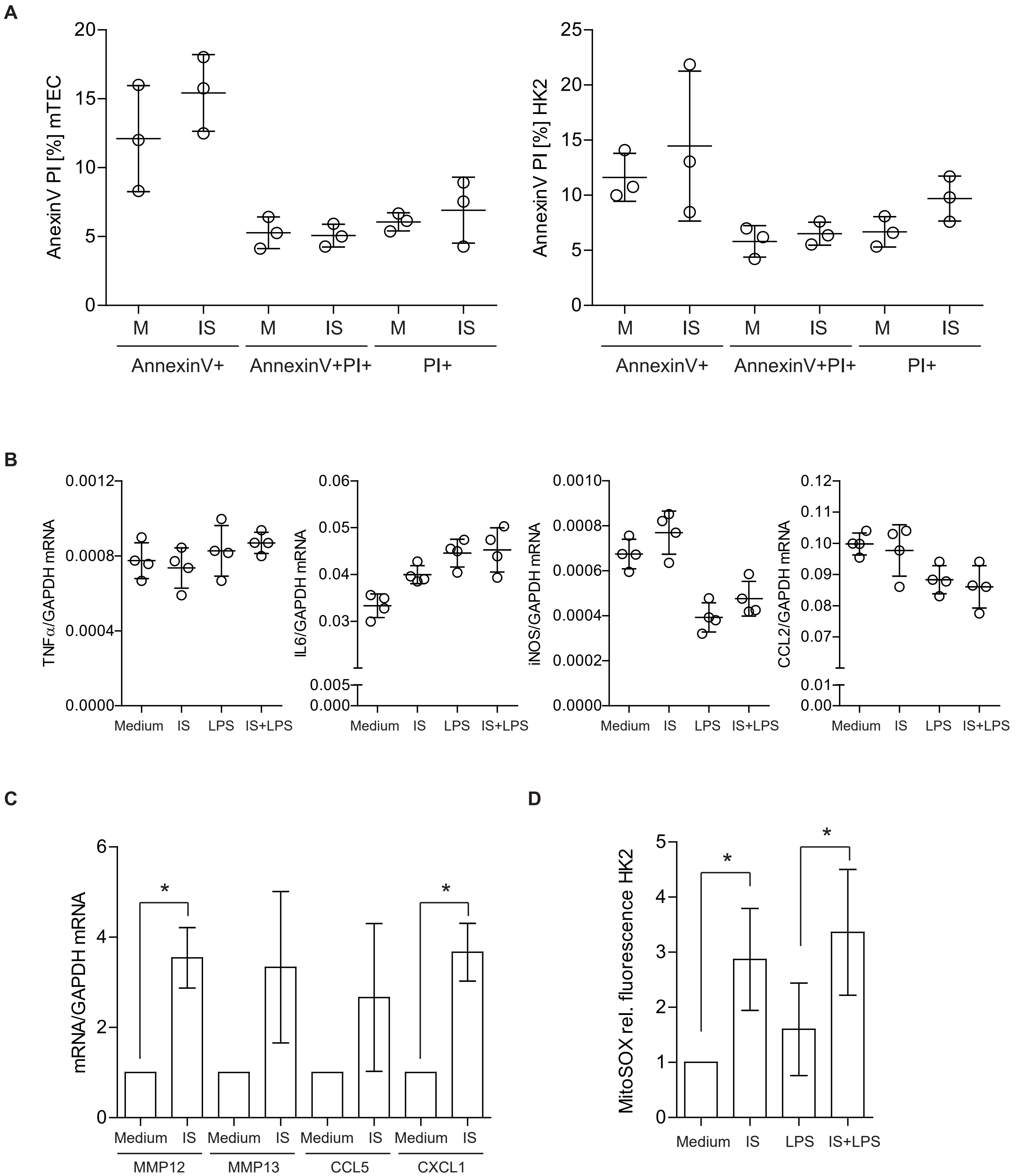

Figure S5. Indoxyl sulfate affects inflammatory responses of tubular epithelial cells.
(A) Annexin V and PI staining of epithelial cells. We cultured the cells in medium containing 60µg/ml indoxyl sulfate (IS) for 24 h. (B) The mRNA expression levels of genes in epithelial cells; we cultured the cells in medium containing 60µg/ml indooxyl sulfate (IS) and stimulated them with LPS (100 ng/mL) for 4h. (C) Figure shows the mRNA expression levels of senescence-associated genes in HK2.
(D) Mitochondrial superoxide production was detected with MitoSOX and presented as MFI fold induction.
Data are shown as means ± SD. * $p < 0.05$.
