# Supplementary material for: Uremic Toxin Indoxyl Sulfate Promotes Macrophage-Associated Low-Grade Inflammation and Epithelial Cell Senescence
Source: Int J Mol Sci. 2023 Apr 28;24(9):8031. doi: 10.3390/ijms24098031 (PMC10179130; doi:10.3390/ijms24098031)
Supplement: Supplementary file 1 [file ijms-24-08031-s001.zip › ijms-2345636-supplementary/Supplementary Figure S7.pdf]

Figure S7

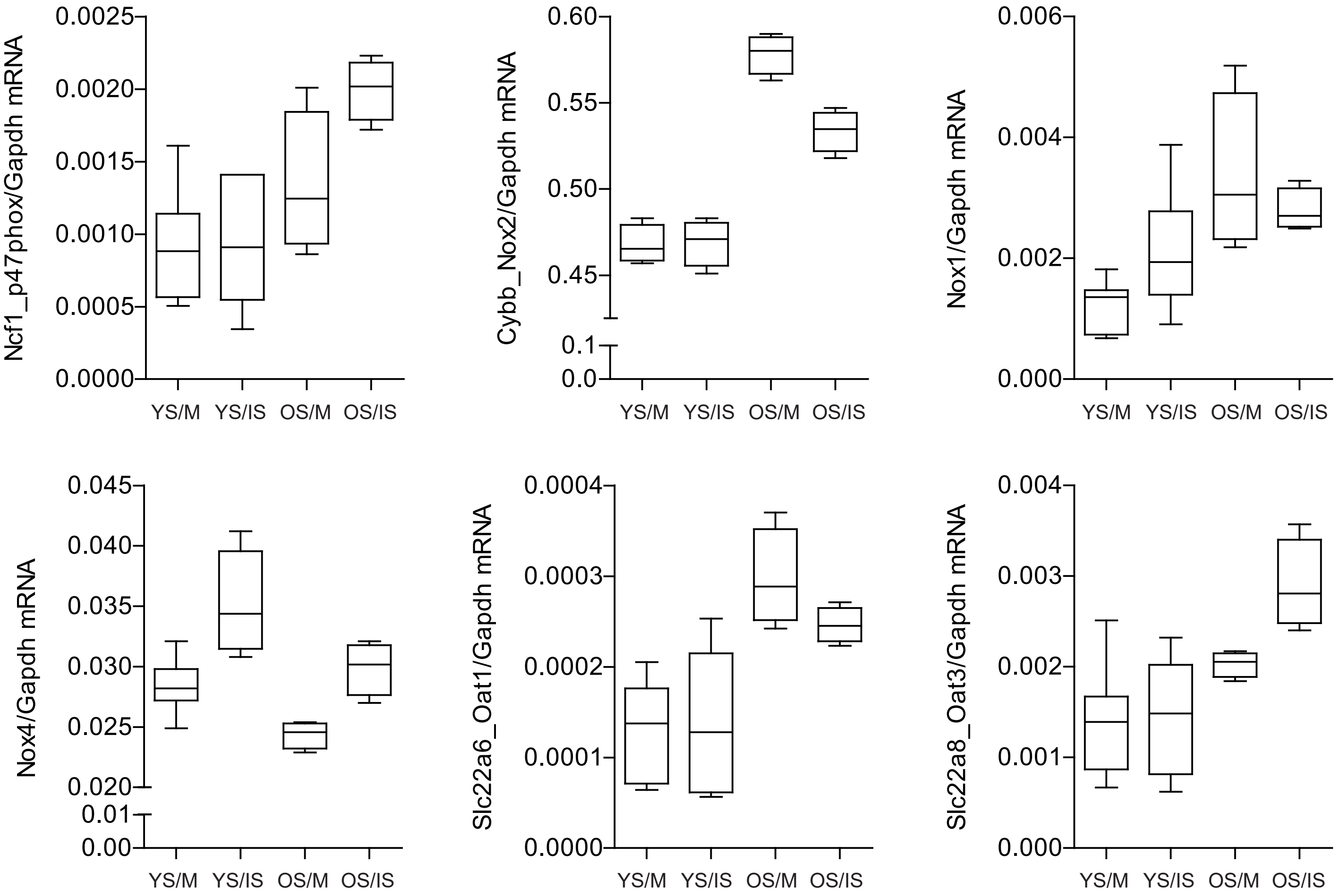

Figure S7. Indoxyl sulfate and ROS-related gene expression in tubular epithelial cells.
Figure shows the mRNA expression levels of ROS-associated genes in tubular epithelial cells
