# Supplementary material for: Uremic Toxin Indoxyl Sulfate Promotes Macrophage-Associated Low-Grade Inflammation and Epithelial Cell Senescence
Source: Int J Mol Sci. 2023 Apr 28;24(9):8031. doi: 10.3390/ijms24098031 (PMC10179130; doi:10.3390/ijms24098031)
Supplement: Supplementary file 1 [file ijms-24-08031-s001.zip › ijms-2345636-supplementary/Supplementary Table S1.pdf]

Supplementary Table S1. Sequence of primers used for RT-PCR studies.

**Primer name**	**Accession Nr.**	**Sequence_Fw**	**Sequence_Rv**
A20	NM_009397	AAGCTCGTGGCTCTGAAAAC	TTCCTCAGGACCAGGTCAGT
Ahr	NM_013464	CTGGTTGTCACAGCAGATGCCT	CGGTCTTCTGTATGGATGAGCTC
Areg	NM_009704	GCAGATACATCGAGAACCTGGAG	CCTTGTCATCCTCGCTGTGAGT
Armcx3	NM_027870	GTGAATGCCGAAAATCAGCGCAG	GGTAAGCAATCTGAGTCCAGCC
Ccl2	NM_011333	GCTACAAGAGGATCACCAGCA	GTCTGGACCCATTCCTTCTTG
Ccl5	NM_013653	CCACTTCTTCTCTGGGTTGG	GTGCCCACGTCAAGGAGTAT
Cdkn1a	NM_007669	GCAGAATAAAAGGTGCCACAGG	AAAGTTCCACCGTTCTCGGG
Cdkn2a	NM_001040654	TGTTGAGGCTAGAGAGGATCTTG	CGAATCTGCACCGTAGTTGAGC
Ctnnb1	NM_001165902	GTTCGCCTTCATTATGGACTGCC	ATAGCACCCTGTTCCCGCAAAG
Cxcl1	NM_008176	ACCCAAACCGAAGTCATAGCC	CTCCGTTACTTGGGGACACC
Cyld	NM_001128170	CACCCTACTGGGAAGAACGGA	ACGGTCTTGGATGTACTGTCCTATA
Dok1	NM_001291799	TTTTCTGCCTTGGAGATGCT	GCTCCAGGATTTGACTCTGC
Dok2	NM_010071	ATGGTCAGGATGGAGGAGC	ATATAACACGGCTGCGAACC
Dsp	NM_023842	TACACCTCAGGGCTGGAAACTC	GTAGTCTCCAGACCTCGTAAGC
Duba	NM_138604	AGCGGGCTACAACAGTGAAG	AAGGCCTTTTCAAACCAGTG
Erbin	NM_001289473	GCCCTGAGACACCCTGAGA	CAACCGCACAAACAAACTTC
Errfi1	NM_133753	GCCTACAATCTGAACTCCCCTG	CCTTGGAGATGGACCACACTCT
Gdf15	NM_011819	AGCCGAGAGGACTCGAACTCA	GGTTGACGCGGAGTAGCAGCT
Glb1	NM_009752	CAAGACAGTGGCTGAAGCTCTG	GAGGAAGCGTTGTTCGGTACAG
Il6	NM_031168	TGATGCACTTGCAGAAAACA	ACCAGAGGAAATTTTCAATAGGC
Il10	NM_010548	ACAGCCGGGAAGACAATAACT	CCTGCATTAAGGAGTCGGTTA
iNos	NM_010927	TTCTGTGCTGTCCCAGTGAG	TGAAGAAAACCCCTTGTGCT
IrakM	NM_028679	CACTGCTGGGAGAGCTTTG	CCAGCCAGCTGTTTGAAAGT
Irf4	NM_013674	TGCAAGCTCTTTGACACACA	CAAAGCACAGAGTCACCTGG
Lancl1	NM_021295	CTCCTGAGTTCTCACATCGCTTG	GGTGGAGGTAAAGCACAGCGAT
Ly6d	NM_010742	CATGCTTTAGCCATGATGGAGGC	ATGTCATCAGCTCTTGGTGCCC
Mcpip1	NM_153159	CCTGTGGTCATCGACGGAAG	GAAGGATGTGCTGGTCTGTGATA
Muc1	NM_013605	AGTGCCTCTGACGTGAAGTCAC	GGGAGGGAACTGCATCTCATTC
Nox4	NM_015760	CGGGATTTGCTACTGCCTCCAT	GTGACTCCTCAAATGGGCTTCC
Otud7b	NM_001025613	GGAGGTGAAGTTACATCTGCTGC	TCAGGAGTGGACCTGGGTTCAT
Pias4	NM_021501	CCAACCGCATTACTGTCACCTG	CGTCTTCAACCTCTGTAGCAGG
Prodh	NM_011172	ATCGCATCCAGGGCTGAGATTG	GTCAATGAGGCTGTTCCAGTCC
S100a9	NM_001281852	TGGTGGAAGCACAGTTGGCAAC	CAGCATCATACACTCCTCAAAGC
Ship	NM_010566	GCTGTTCCGGAATTGTGTTT	GTGAAGAACCTCATGGGGAC
Sigirr	NM_023059	AGAGGTCCCAGAAGAGCCAT	ACACTGGGGCCTAGAGAACA
Sike1	NM_025679	TTCAGGTGGACGATAACCAA	GAGATTCACTGCTGATGGACAG
Slc9a3r1	NM_012030	ATCTGCCTCCAGCGATACCAGT	AGCCAAGGAGATGTTGAGGTCC
Slc22a6	NM_008766	GAAAGGCTGTCTGGCTTCCTCT	ATCAGTGGGCTCACTATGCTGC
Slc22a8	NM_031194	AGGCTCTGAAGACACTCCAACG	ACCTTGGCTGAGGTGATGTCCT
Smurf2	NM_025481	CCAATGCCATCAACCGCCTCAA	GTGCCTATTCGGTCTCTGGACT
Snx14	NM_172926	GTGGCTGAAGGCGAAGATGACT	CGAGGAGTGTTAGGTGTGCTCA
Socs1	NM_001271603	ACTTCTGGCTGGAGACCTCA	ACAAGCTGCTACAACCAG
Socs3	NM_007707	AAGGCCGGAGATTTCGCT	AACTTGCTGTGGGTGACCAT
Stx4a	NM_009294	GTCCCAGCAATTTGTCGAGCTC	TCCTCGTCAGACACCATTCCAG
Tank	NM_001164071	GCTTCCAGAATGGGTACGTG	TGGTAGGAATGCCAGCTCTC
Tgfb	NM_011577	GGAGAGCCCTGGATACCAAC	CAACCCAGGTCCTTCCTAAA
Tnfa	NM_013693	AGGGTCTGGGCCATAGAACT	CCACCACGCTCTTCTGTCTAC
Tollip	NM_001347562	GCGGGTCTCTGTGCAGTT	TGTGGGTGTTATACGGAGGAA
Trim32	NM_001161782	GAAACAGCGGAGTTCTGAGGCA	AACCAGCACCTCACTCTGACTG

Tslp	NM_021367	GCAAATCGAGGACTGTGAGAGC	TGAGGGCTTCTCTTGTTCTCCG
Twist1	NM_011658	GATTCAGACCCTCAAACTGGCG	AGACGGAGAAGGCGTAGCTGAG
Vamp3	NM_009498	AGACCAGAAGCTCTCGGAGCTA	ACCAGGACACTGATCCCTATCG
Zranb1	NM_207302	GGCTATTCCTCAATGCCTGTGTG	AGAAGGACGGTTCAGCAAGCGT
hA20	NM_001270507	GGACTTTGCGAAAGGATCG	TCACAGCTTTCCGCATATTG
hCCL2	NM_002982	AGAATCACCAGCAGCAAGTGTCC	TCCTGAACCCACTTCTGCTTGG
hCCL5	NM_002985	CCTGCTGCTTTGCCTACATTGC	ACACACTTGGCGGTTCTTTCGG
hCXCL1	NM_001511	AGCTTGCCTCAATCCTGCATCC	TCCTTCAGGAACAGCCACCAGT
hIL-6	NM_000600	AGTGAGGAACAAGCCAGAGC	GTCAGGGGTGGTTATTGCAT
hiNOS	NM_000625	GCTCTACACCTCCAATGTGACC	CTGCCGAGATTTGAGCCTCATG
hMCPIP1	NM_025079	AAGAGGAAAAGGAGGGCAGC	ATGTGCTGGTCTGTGATGGG
hTNFa	NM_000594	CTCTTCTGCCTGCTGCACTTTG	ATGGGCTACAGGCTTGTCACTC
