# Supplementary figures and images for: Uremic Toxin Indoxyl Sulfate Promotes Macrophage-Associated Low-Grade Inflammation and Epithelial Cell Senescence
Source: Int J Mol Sci. 2023 Apr 28;24(9):8031. doi: 10.3390/ijms24098031 (PMC10179130; doi:10.3390/ijms24098031)

Figure S4

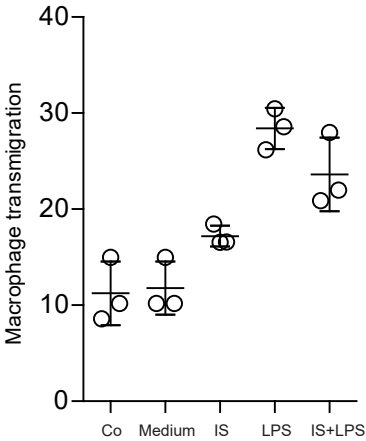

Figure S4. Indoxyl sulfate does not affect transmigration of BMDM.

Supplement: Supplementary file 1 [file ijms-24-08031-s001.zip › ijms-2345636-supplementary/Supplementary Figure S4.pdf]
